# The Herald of Health

**Published:** 1873-12

**Authors:** 


*The Herald of Health.* Now in the twenty second volume of its new series, continues its endeavors to improve body and mind by advocating a higher manhood, physical, intellectual and moral. The contents for December, 1873, are Rights of Children, A Scamper across Europe, Our Food, Wisdom Crumbs, True Beauty, Disease Propagated by Milk, Lessons for the Children, Editor's Studies in Hygiene, Desert Table, and Topics of the Mouth.

Published by Wood & Holbrook, 13 and 15 Laight Street, New York, $1 50 per annum. Single copy 15 cents.

